# Clinical epidemiology of the endoscopic, laparoscopic, and surgical resection of malignant gastric tumors in Japan, 2014–2021: a retrospective study using open data from a national claims database

**DOI:** 10.1007/s10120-024-01553-y

**Published:** 2024-09-28

**Authors:** Akahito Sako, Tomoyuki Yada, Keiichi Fujiya, Ryo Nakashima, Kensuke Yoshimura, Hidekatsu Yanai, Naomi Uemura

**Affiliations:** 1 Department of Internal Medicine, Kohnodai Hospital, National Center for Global Health and Medicine, 1-7-1 Kohnodai, Ichikawa, Chiba, 272-8516 Japan; 2 Department of Gastroenterology and Hepatology, Kohnodai Hospital, National Center for Global Health and Medicine, 1-7-1 Kohnodai, Ichikawa, Chiba, 272-8516 Japan; 3 Division of Gastric Surgery, Shizuoka Cancer Center, 1007 Shimonagakubo, Nagaizumi-cho, Sunto-gun, Shizuoka, 411-8777 Japan; 4 Department of Gastroenterological Surgery, Fukuoka University Faculty of Medicine, 7-45-1 Nanakuma, Jonan-ku, Fukuoka, 814-0133 Japan; 5 Center for Next Generation of Community Health, Chiba University Hospital, 1-8-1 Inohana, Chuo-ku, Chiba, 260-8677 Japan

**Keywords:** Gastric cancer, Endoscopic submucosal dissection, Gastrectomy, Regional disparity, Real-World data

## Abstract

**Background:**

Gastric cancer is a common malignancy with a high incidence in East Asia. Gastric resection ranges from endoscopic resection to open total gastrectomy. However, nationwide data are lacking.

**Methods:**

This observational study analyzed data from the publicly accessible National Database of Health Insurance Claims and Specific Health Checkups, which includes most national health insurance claims data in Japan. Trends in the types of resection performed for malignant gastric tumors between 2014 and 2021, patients’ age and sex distributions, and regional disparities were investigated.

**Results:**

The annual number of resections was highest in 2015 (109,000) and lowest in 2020 (90,000) after the COVID-19 pandemic. The proportion of endoscopic resections increased from 47% in 2014 to 57% in 2021 while that of total gastrectomies decreased from 17 to 10%. In 2021, 70% of patients who underwent resection were men. That year, 83.8% of all patients who underwent any type of gastric resection and 87.1% of those who underwent endoscopic submucosal dissection were aged ≥ 65 years. The annual incidence of gastric resection per million population was highest in Tottori (*n* = 1236) and lowest in Okinawa (*n* = 251). The proportion of endoscopic resections was highest in Miyagi (66%) and lowest in Aichi (45%) and that of open surgery was highest in Aomori (36%) and lowest in Wakayama (5%).

**Conclusions:**

Gastric malignancy is increasingly treated by endoscopic submucosal dissection rather than open total gastrectomy. However, regional disparities remain in resection type. Standardization of treatment and a more even distribution of specialists are needed.

**Supplementary Information:**

The online version contains supplementary material available at 10.1007/s10120-024-01553-y.

## Background

Gastric cancer is one of the most common malignancies worldwide and has a high incidence in East Asia and South America due to their higher prevalence of *Helicobacter pylori* infection [1, 2]. Prompt diagnosis of gastric cancer is important because of the higher likelihood of curative treatment in the early stages and the greater opportunity to use less invasive treatment options, such as endoscopic submucosal dissection (ESD).

In Japan, the Cancer Control Act enacted in 2007 identified the basic means of cancer control to be consistency of treatment, prevention and early diagnosis of cancer, and promotion of cancer research. In Japan, there are regional disparities in the rate of *H. pylori* infection and in the incidence and mortality of gastric cancer [3, 4]. However, data on the treatment of gastric cancer according to region are limited. Detailed nationwide or large-scale data on endoscopic and surgical resection of gastric malignancy are lacking in many countries, including Japan. The National Clinical Database (NCD) contains nationwide data on most of the surgeries performed in Japan, including patient characteristics, indications for and types of surgery, and morbidity and mortality [5–9], but does not include endoscopic resection.

In this descriptive study, we investigated the clinical epidemiology of endoscopic and surgical resection of malignant gastric tumors in Japan by analyzing the nearly complete enumeration in the dataset held by a national health insurance claims database.

## Methods

This retrospective cohort study analyzed open data from the National Database of Health Insurance Claims and Specific Health Checkups (NDB) of Japan. Japan has a population of 126 million and a universal health care system. The Ministry of Health, Labour and Welfare has used the NDB to collect almost all national health insurance claims data for both inpatients and outpatients since 2009. The NDB contains data on patient sex, age, diagnosis, examinations, prescriptions, and surgeries. NDB Open Data, an anonymized dataset extracted from the NDB and made publicly available on the NDB website, does not provide individual data; it provides aggregate data, including annual numbers of gastric surgeries and upper endoscopic examinations performed in Japan stratified by sex, 5-year age intervals, inpatient/outpatient status, month, and geographic location of clinics or hospitals in Japan’s 47 prefectures (from Hokkaido in the north to Okinawa in the south) [10]. Age distribution by sex is also available, but the age distribution within each prefecture is not. No diagnoses are available. In Japan, the fiscal year (FY) runs from April 1 to March 31, and as of December 2023, data are available for FY2014 to FY2021. We analyzed data for this entire period, with a focus on FY2021 as the most recent data available. For the denominator, we used the population of Japan stratified by age, sex, and prefecture, as reported by the Statistics Bureau of Japan [11].

To investigate the influence of COVID-19 on gastric resections, we considered the monthly number of gastric resections and COVID-19 cases [12], the start of vaccinations, the state of emergency, and the first documented cases of COVID-19 caused by the Delta and Omicron variants in Japan between FY2019 and FY2021. We evaluated the change in the monthly number of gastric resections from the average in FY2019 as a reference standard.

To consider the age distribution and the regional differences in gastric cancer-screening rate and regional differences in mortality, we used data from the National Cancer Center [3] and the Statistics Bureau of Japan [11]. The cancer-screening rate was based on the Comprehensive Survey of Living Conditions by the Ministry of Health, Labour, and Welfare, conducted in 2022. It is a nationwide questionnaire survey of a stratified random sample and asks whether the respondent has participated in gastric cancer screening in the past year. In Japan, barium examination or upper endoscopy is offered as part of the annual health checkups provided by municipalities and employers, with the aim of screening for gastric cancer. In NDB Open Data, upper endoscopic examination does not include upper endoscopy performed as part of a cancer-screening program.

To analyze the regional differences in terms of the number and choice of surgeries, we used the number of the board-certified fellows of the Japan Gastroenterological Endoscopy Society, board-certified surgeons in gastroenterology (the Japanese Society of Gastroenterological Surgery), endoscopic surgical skill qualification system–qualified gastric surgeons (Japan Society for Endoscopic Surgery), and RoboDoc-certified gastroenterologists (Japan Robotic Surgery Society) per million general population in each prefecture in 2024. The number of specialists in 2021 was not available. We collected the number of specialists from the website of these societies.

Data from NDB Open Data are anonymized, so there are some missing values in the data. Data are available in the form of an Excel spreadsheet for the total annual number of surgeries and distribution by sex, 5-year age interval, and prefecture. However, if the number of surgeries in a specific cell is low, the data are masked. The cut-off value for masking is 10. For example, if only 8 ESDs were performed for men aged 20–24 years in 2021, this figure would be masked.

Many studies have examined data from the NDB [13, 14] and NDB Open Data [15–17]. The institutional review board of the National Center for Global Health and Medicine reviewed and approved the study protocol. The need for informed consent was waived because the data in NDB Open Data are anonymized and publicly available.

### Endoscopic, laparoscopic, and surgical resection of malignant gastric tumors

For the purposes of the Japanese health reimbursement system, endoscopic resection for malignant gastric tumors is divided into polypectomy, endoscopic mucosal resection (EMR), and ESD for early-stage malignant tumors of the stomach and duodenum. ESD for the stomach and ESD for the duodenum have been differentiated since 2020. We did not include endoscopic resection for non-malignant tumors. Local gastrectomy is divided into open, laparoscopic, and laparoscopic and endoscopic collaborative surgery (LECS). LECS for the duodenum has been differentiated from LECS for the stomach since 2020. The extent of gastric resection was divided into six groups, namely, EMR and polypectomy, ESD, local gastrectomy, distal gastrectomy, proximal gastrectomy, and total gastrectomy. The types of gastric resections were categorized as LECS, endoscopic, robot-assisted, laparoscopic, and open surgery. Robot-assisted gastrectomy was first approved for Advanced Medical Technology in 2014 and first covered by public health insurance and included in the NDB in 2018. Gastrectomy for non-malignant disease was excluded.

## Results

### Annual numbers and types of resections for malignant gastric tumors

Between 2014 and 2019, the annual number of resections performed for malignant gastric tumors gradually decreased from 109,000 to 103,000, which coincided with a decrease in the number of upper endoscopies from 9,340,000 to 8,586,000 (Table 1). The proportion of endoscopic resections increased from 46.9% in 2014 to 56.5% in 2021. ESD accounted for 92%–93% of endoscopic resections. The proportion of laparoscopic surgeries increased from 19.8% in 2014 to 24.4% in 2021. Robot-assisted surgery accounted for 5.5% of all laparoscopic surgeries performed in 2018 and 16.8% of those performed in 2021. The proportion of total gastrectomies performed decreased from 16.5% in 2014 to 10.0% in 2021.

**Table 1:** Annual numbers of gastric resections and upper endoscopies performed between 2014 and 2021

Fiscal year	2014	2015	2016	2017	2018	2019	2020	2021
Polypectomy for stomach and duodenum	459	401	422	441	410	434	370	457
	0.4%	0.4%	0.4%	0.4%	0.4%	0.4%	0.4%	0.5%
EMR for stomach and duodenum	3365	3123	3075	3054	3139	3348	2996	3342
	3.1%	2.9%	2.9%	2.9%	3.0%	3.2%	3.3%	3.5%
ESD for stomach and duodenum	47,045	48,737	49,257	50,975	51,603	53,386		
	43.4%	44.9%	46.7%	48.4%	50.0%	51.1%		
ESD for stomach							45,520	49,197
							50.5%	52.0%
ESD for duodenum							422	456
							0.5%	0.5%
Open local gastrectomy	616	629	672	614	606	594	528	563
	0.6%	0.6%	0.6%	0.6%	0.6%	0.6%	0.6%	0.6%
Laparoscopic local gastrectomy	941	979	1044	1176	1218	1407	1217	1358
	0.9%	0.9%	1.0%	1.1%	1.2%	1.3%	1.3%	1.4%
LECS for stomach	918	1115	1190	1312	1376	1497	1248	1444
	0.8%	1.0%	1.1%	1.2%	1.3%	1.4%	1.4%	1.5%
LECS for duodenum							114	171
							0.1%	0.2%
Open distal gastrectomy	19,269	18,608	16,408	14,973	13,206	11,927	9974	9049
	17.8%	17.1%	15.6%	14.2%	12.8%	11.4%	11.1%	9.6%
Laparoscopic distal gastrectomy	16,184	16,291	16,337	16,678	15,872	15,829	13,286	13,704
	14.9%	15.0%	15.5%	15.8%	15.4%	15.2%	14.7%	14.5%
Robot-assisted distal gastrectomy					901	1693	2036	2622
					0.9%	1.6%	2.3%	2.8%
Open proximal gastrectomy	969	1005	954	918	814	797	676	615
	0.9%	0.9%	0.9%	0.9%	0.8%	0.8%	0.7%	0.7%
Laparoscopic proximal gastrectomy	814	932	1103	1386	1448	1725	1355	1489
	0.8%	0.9%	1.0%	1.3%	1.4%	1.7%	1.5%	1.6%
Robot-assisted proximal gastrectomy					153	324	408	606
					0.1%	0.3%	0.5%	0.6%
Open total gastrectomy	14,398	13,512	11,697	10,561	9167	8168	6836	6184
	13.3%	12.4%	11.1%	10.0%	8.9%	7.8%	7.6%	6.5%
Laparoscopic total gastrectomy	3478	3272	3229	3229	3067	2879	2621	2639
	3.2%	3.0%	3.1%	3.1%	3.0%	2.8%	2.9%	2.8%
Robot-assisted total gastrectomy					205	388	543	644
					0.2%	0.4%	0.6%	0.7%
Total number of gastric resections	108,456	108,604	105,388	105,317	103,185	104,396	90,150	94,540
Upper endoscopic examinations	9,044,848	9,339,686	8,958,531	8,806,118	8,740,469	8,585,843	7,532,324	7,936,693

*EMR* endoscopic mucosal resection; *ESD* endoscopic submucosal dissection; *LECS* laparoscopic and endoscopic collaborative surgery

### Age and sex distribution

In 2021, 69.7% of patients who underwent resection of malignant gastric tumors were men. Although men accounted for 60%–80% of most types of resections, similar numbers of men and women underwent local gastrectomy, including LECS.

Patients aged ≥ 65 years and ≥ 75 years, respectively accounted for 83.8% and 48.7% of all types of gastric resection and 87.1% and 52.0% of ESDs performed for malignant gastric tumors.

**Fig. 1:**
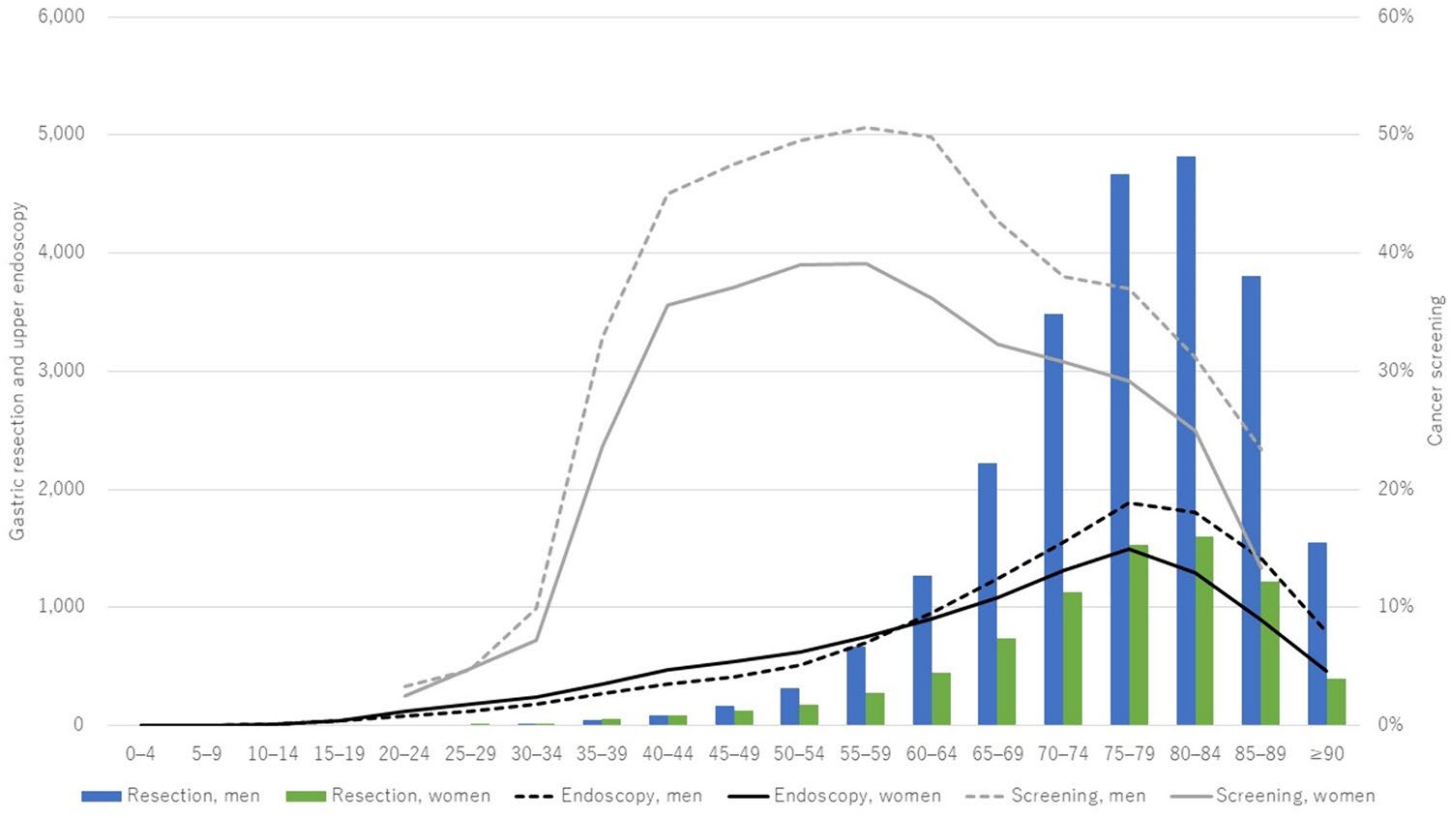
Numbers of gastric resections per million general population and upper endoscopies per 10,000 general population performed in 2021 and gastric cancer screening rate in 2022 by age and sex

The incidence of upper endoscopic examination was highest in patients aged 75–79 years, irrespective of sex (Fig. 1). The annual incidence of upper endoscopy per 10,000 general population aged 75–79 years was 1888 for men and 1493 for women. Specifically, 1 in 5.3 men and 1 in 6.7 women aged 75–79 years underwent upper endoscopy annually. The gastric cancer-screening rate was highest in men (50.6%) and women (39.1%) aged 55–59 years in 2022.

The incidence of gastric resection was highest in patients aged 80–84 years irrespective of sex (Fig. 1). The annual incidence of gastric resection per million general population aged 80–84 years was 4819 for men and 1599 for women.

**Fig. 2:**
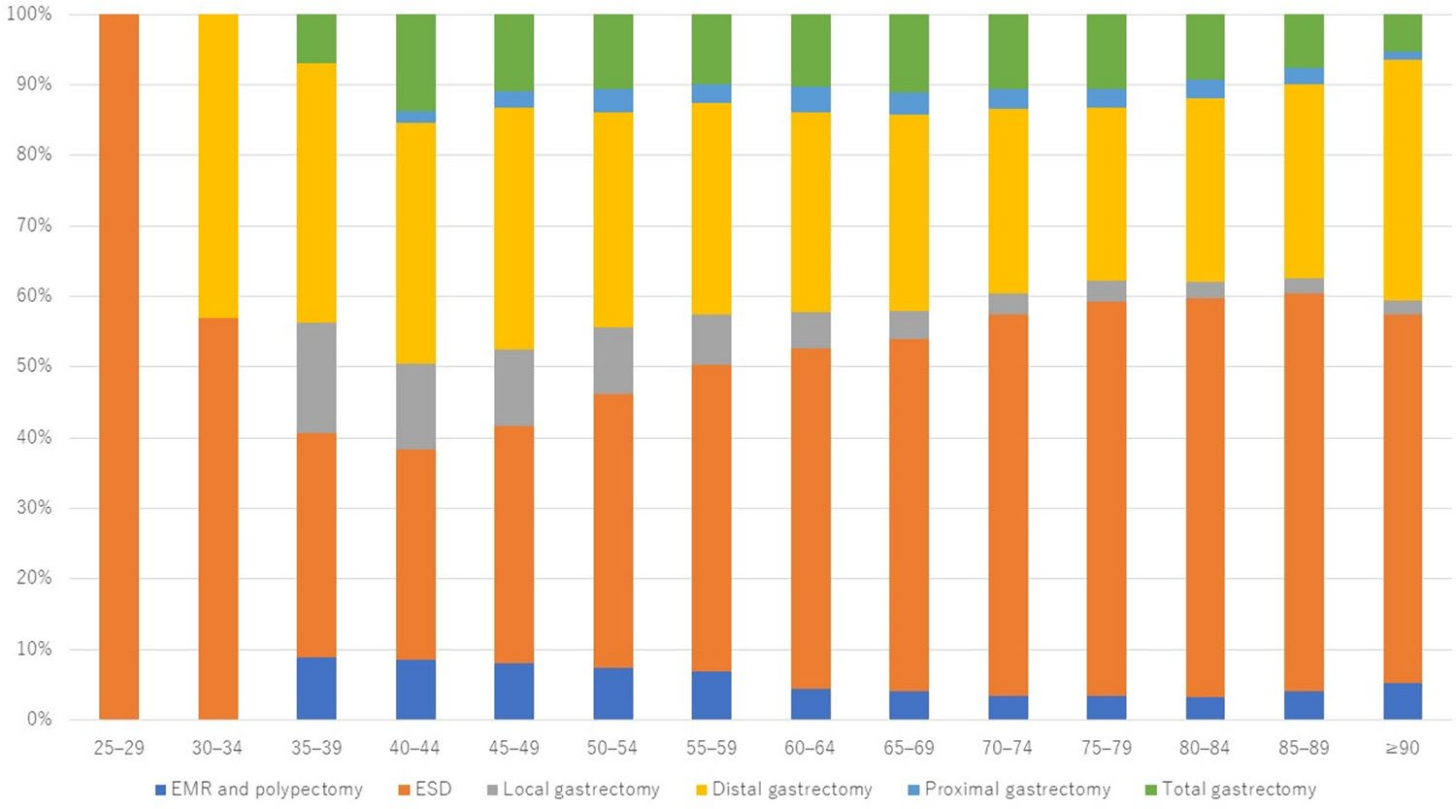
Extent of gastric resections performed in 2021 by age. EMR, endoscopic mucosal resection; ESD, endoscopic submucosal dissection

In terms of extent of resection, the proportions of endoscopic resection, LECS and local gastrectomy, distal gastrectomy, proximal gastrectomy, and total gastrectomy were 56.8%, 3.6%, 26.9%, 2.8%, and 10.0%, respectively (Fig. 2). LECS and local gastrectomy were more common in younger patients. The older the patient, the more likely they were to undergo endoscopic resection and the lower the likelihood of total gastrectomy.

**Fig. 3:**
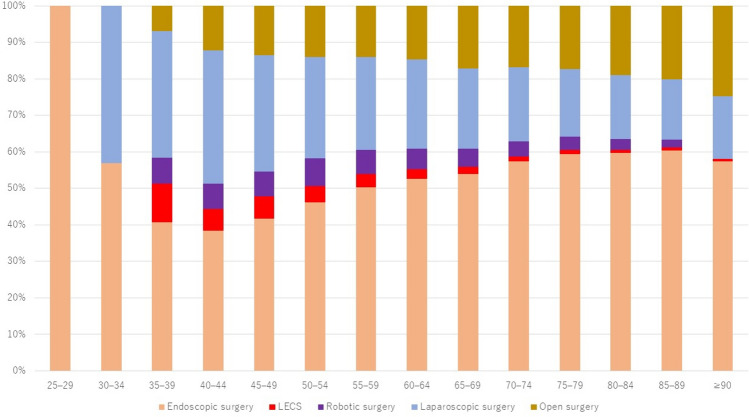
Types of gastric resection performed in 2021 by age

In regard to the resection procedures performed, the respective proportions of endoscopic resection, LECS, robot-assisted surgery, laparoscopic surgery, and open surgery were 56.8%, 1.6%, 4.0%, 20.3%, and 17.3% (Fig. 3). Open surgery was performed predominantly in older patients and laparoscopic and robot-assisted surgery in relatively young patients.

### Geographic differences by prefecture

**Fig. 4:**
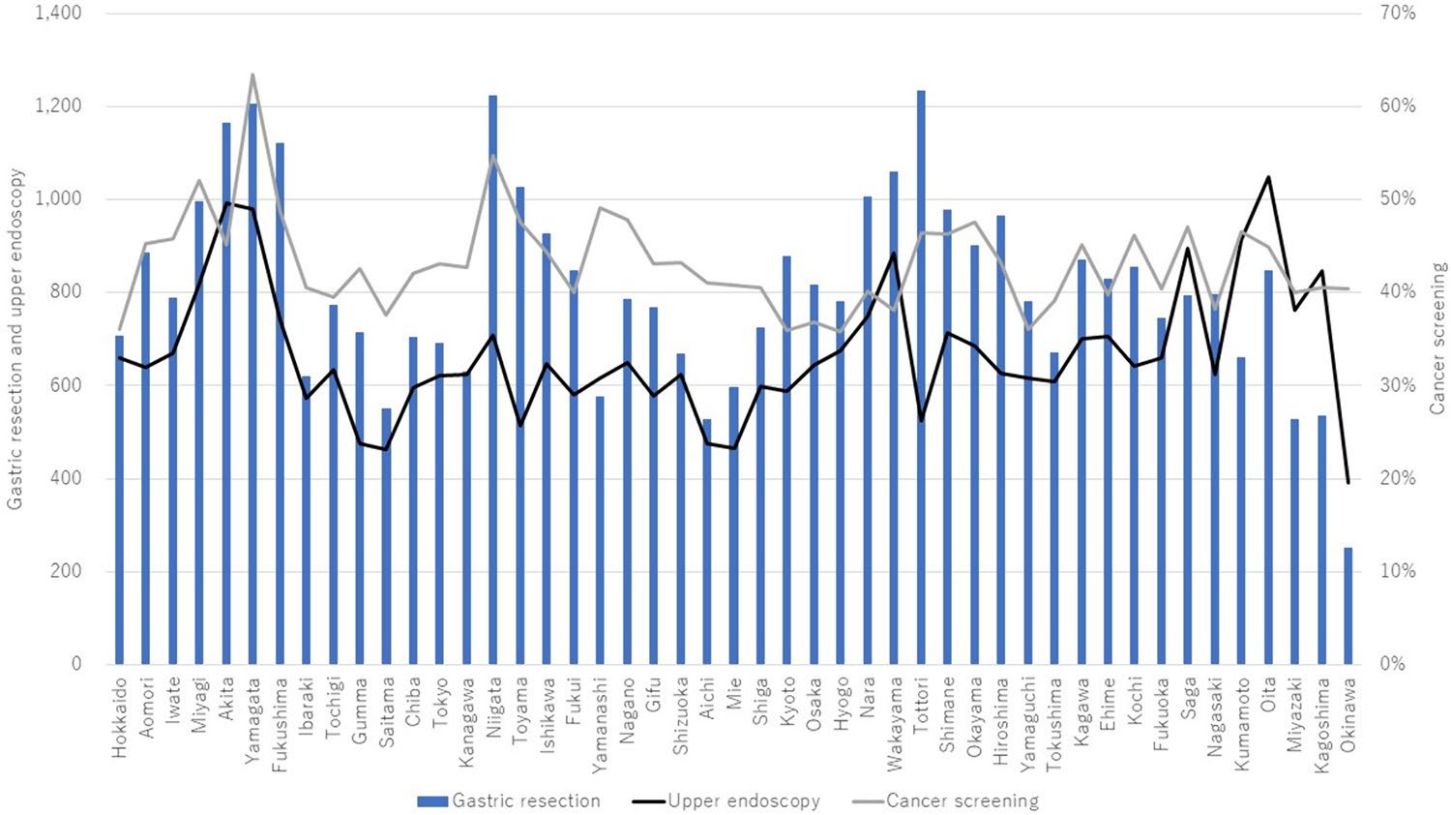
Numbers of gastric resections per million general population and upper endoscopies per 10,000 general population performed in 2021 and gastric cancer screening rate in 2022 by prefecture

In 2021, the incidence of upper endoscopy per 10,000 general population was highest in Oita (1051), followed by Akita (993) and Yamagata (979), and was lowest in Okinawa (391) followed by Saitama (462) and Mie (466) (Fig. 4).

In 2022, the gastric cancer-screening rate was highest in Yamagata (63.5%), followed by Niigata (54.7%) and Miyagi (52.1%), and was lowest in Hyogo (35.8%), followed by Kyoto (35.9%), Hokkaido (36.1%), and Yamaguchi (36.1%).

In 2021, the incidence of gastric resection per million general population was 753 in Japan. It was highest in Tottori (1236), followed by Niigata (1225) and Yamagata (1207), and was lowest in Okinawa (251) followed by Miyazaki (528) and Aichi (529).

**Fig. 5:**
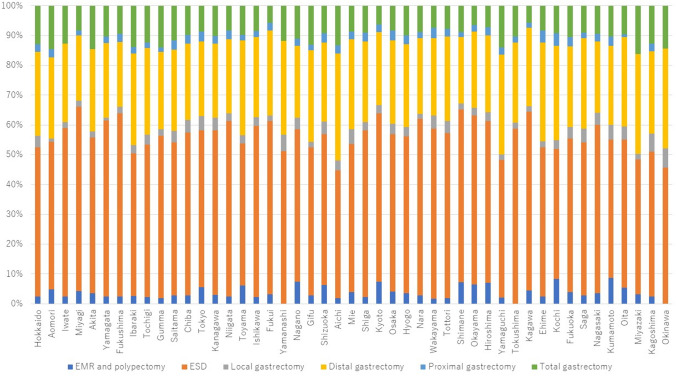
Extent of gastric resections performed in 2021 by prefecture. EMR, endoscopic mucosal resection; ESD, endoscopic submucosal dissection

In terms of extent of resection, the proportion of patients who underwent total gastrectomy was highest in Miyazaki (16.3%), followed by Akita and Aomori (14.6%), and was lowest in Kagawa and Fukui (5.7%), followed by Kyoto (6.3%) (Fig. 5). The proportion of patients who underwent endoscopic resection was highest in Miyagi (66.2%), followed by Shimane (65.3%) and Kagawa (64.4), and was lowest in Aichi (44.7%), followed by Okinawa (45.7%) and Yamaguchi (48.3%).

**Fig. 6:**
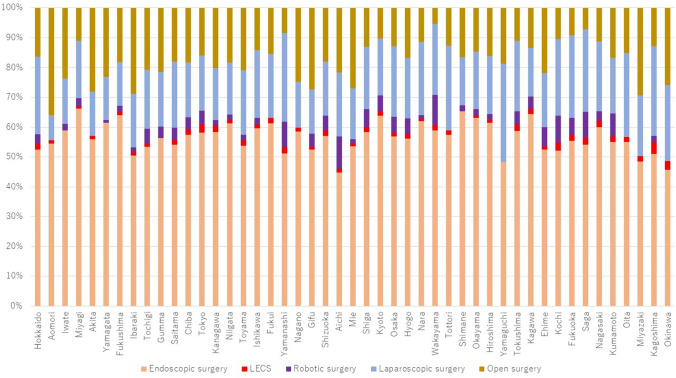
Types of gastric resections performed in 2021 by prefecture

With regard to type of resection, open surgery was most common in Aomori (36.0%), followed by Miyazaki (29.3%) and Ibaraki (28.8%), and was lowest in Wakayama (5.3%), followed by Saga (7.2%) and Yamanashi (8.4%) (Fig. 6). Robot-assisted surgery was performed most often in Aichi (10.9%), followed by Wakayama (10.1%) and Kochi (8.9%), and 0% in 9 prefectures.

In 2024, the number of gastroenterological specialists per million general population is as follows (Online Resource 1). The number of the board-certified fellows of the Japan Gastroenterological Endoscopy Society was highest in Oita (222.5) and lowest in Okinawa (101.5). The number of the board-certified surgeons in gastroenterology was highest in Tottori (107.5) and lowest in Ibaraki (46.3). The number of the endoscopic surgical skill qualification system-qualified gastric surgeons was highest in Tottori (16.4) and lowest in Aomori (0.8). The number of RoboDoc-certified gastroenterologists was highest in Ehime (12.1) and lowest in Nagano (0).

In 2021, the age-standardized mortality rate for gastric cancer under the age of 75 years per 100,000 general population in men was highest in Aomori (13.8), followed by Tottori (13.7) and Akita (12.8), and was lowest in Yamanashi (6.5), followed by Nagano (7.0) and Kumamoto (7.2) (Online Resource 2); that in women was highest in Aomori (5.8) and Saga (5.8), followed by Yamagata (5.3), and was lowest in Kumamoto (2.5), followed by Nagano (2.7), Tokushima (2.8), and Okinawa (2.8).

### COVID-19 pandemic and monthly number of gastric resections

Compared with FY 2019, the annual number of upper endoscopies, endoscopic resections, and gastric resections except for endoscopic resection in FY2020 decreased to 87.7%, 86.3%, and 86.5%, respectively (Table 1). In FY 2021 these showed some recovery to 92.4%, 93.5%, and 87.0%. The monthly number of gastric resections and upper endoscopic examinations fluctuated along with the change in the number of COVID-19 cases and the state of emergency (Online Resources 3 and 4). The first documented case of COVID-19 in Japan was in January 2020. The largest drop in both surgeries and upper endoscopies was seen in May 2020, during the first state of emergency. At first, the number of upper endoscopic examinations decreased to around 89% of the average for FY 2019 between January and March 2020 and decreased further to 61.7% in April and 53.3% in May before recovering to 88.9% in June. At the same time, the number of endoscopic resections decreased to 89.7% in April and 64.5% in May before recovering to 75.4% in June. The number of gastric resections except for endoscopic resections decreased to 96.4% in April and 77.9% in May before recovering to 80.4% in June.

## Discussion

This nationwide retrospective study investigated trends in age and sex distribution as well as regional disparities in resection of malignant gastric tumors in Japan during the 8 years between 2014 and 2021. About 100,000 resections were performed annually; 48.7% were performed in patients aged ≥ 75 years and 69.7% were men. Large regional disparities in the incidence and type of gastric resection were identified.

The NCD collects surgical data in Japan. The majority of surgical procedures for malignant gastric tumors, including laparoscopic surgery, are included in this database. Several studies of gastric cancer surgery have used the NCD to obtain information on intra-abdominal infections after surgery, long-term survival, surgery-related mortality, and comparisons of laparoscopic and robot-assisted surgery [5–9]. However, the NCD does not contain data on endoscopic surgeries, such as ESD. The Japan Gastroenterological Endoscopy Society started the Japan endoscopy database (JED) in 2015 to collect information on endoscopic examinations and treatments [18]. However, its coverage is insufficient in terms of the numbers of participating facilities and endoscopic procedures performed. Moreover, there are still no published studies of gastric cancer based on data from the JED. Although a national cancer registry system was established in Japan in 2016 [19], it is still not widely used and there are currently no published papers concerning gastric cancer that have used data from this registry. Other administrative claims databases include the NDB [13] and the Diagnosis Procedure Combination database [20, 21]. These databases, unlike the NCD and JED, need less workload for data registration and allow investigation of both surgical and endoscopic procedures. For better clinical practice and evidence-based policy-making, it is important to contribute to these databases and use the data effectively.

Between FY2014 and FY2019, there was a gradual decrease in both the number of malignant gastric tumor resections and the number of upper gastrointestinal endoscopies performed, possibly due to *H. pylori* eradication therapy for peptic ulcer becoming covered by health insurance in FY2002 and *H. pylori*-infected gastritis being included in FY2012 [22, 23]. Over time, the numbers of total gastrectomies and open gastrectomies have decreased, with those of open total gastrectomies and open distal gastrectomies having decreased by about half. In the meantime, the frequency of endoscopic resection has increased. Moreover, in 2021, robot-assisted surgery accounted for 19.6% of laparoscopic total gastrectomies and 16.1% of distal gastrectomies. The Japanese guidelines for treatment of gastric cancer expanded the indications for ESD in 2018 and 2021 and added a recommendation for robot-assisted surgery in 2021 [24]. These changes in the clinical guidelines and population aging are possible reasons for the increase in cases that are treated less invasively and the reduction in open and total gastrectomies performed. However, data from NDB Open Data, which do not include detailed information, cannot explain the changes in treatment that have occurred over time.

Studies investigating the influence of COVID-19 on health services related to gastric cancer in Japan reported that gastric cancer was more greatly affected by COVID-19 compared with other cancers [25–27]. In 2020, participants of gastric cancer screening provided by municipalities decreased by 26.1% compared with 2017–2019 [25]. A multicenter questionnaire survey conducted in the Tohoku region revealed that the number of upper endoscopies decreased by 10.1% and the number of esophageal and gastric cancer detected by endoscopy decreased by 5.1% in 2020 compared with 2019 [26]. A nationwide study using NCD reported that the monthly number of distal gastrectomies for gastric cancer between May and December 2020 decreased by 19% compared with that between 2018 and April 2020, and the decline was larger for earlier-stage cancers [27]. The present study demonstrated the nationwide influence of the COVID-19 pandemic on gastric resections of all types as well as upper endoscopy. In May 2020, upper endoscopy and endoscopic resection decreased more than surgical resection did. This difference might be because patients undergoing surgical resection tended to have more advanced gastric cancer. Although the monthly number of endoscopies and endoscopic resections was affected by COVID-19 to a greater extent compared with surgical resection, the decrease in the annual number was similar. This suggests that endoscopic resections were initially delayed, but these delays were handled and the backlog was resolved due to the adjustment of clinical practices and the introduction of preventive measures [28, 29]. The present study did not find a relative decrease in endoscopic resections or a relative increase in total gastrectomies due to the delay of cancer detection. These procedures might have been offset by the increasing trend in endoscopic resections and the decreasing trend in total gastrectomies before COVID-19 pandemic.

We found that more men underwent gastric cancer screening, upper endoscopy, and gastric resection during the study period, which is consistent with the fact that gastric cancer is more common in men and the elderly [2]. Despite the higher gastric cancer-screening rate and more opportunities for upper endoscopies, men have a higher incidence of gastric resection and higher mortality for gastric cancer compared with women. Thus, efforts should be made to promote and facilitate gastric cancer screening for men. The relatively balanced sex distribution and predominance of local gastrectomy, including LECS, in non-elderly patients is thought to reflect the characteristics of gastrointestinal stromal tumors (GISTs) [30], which is a principal target for such procedures. The majority of patients who need endoscopic or surgical resection for gastric malignancy are elderly. The older patients were, the more likely they were to undergo ESD and the less likely they were to under total gastrectomy. This might be due to the early detection of cancer and the difficulty of performing invasive surgery in elderly patients. Although our study does not have detailed and individual information such as clinical stage and the means by which cancer was detected, easy access to upper endoscopy and frequent provision, especially for elderly patients, might have contributed to the observed outcome.

The present study is the first to show regional disparities in the choice of resection procedures for malignant gastric tumors. Previous studies using NDB Open Data have shown regional disparities in clinical practice for nephrectomy and nephroureterectomy [17] as well as prescriptions for influenza and chronic kidney disease [15, 16]. Differences in age distribution, prevalence and strains of *H. pylori*, and in the rate of participation in cancer screening lead to differences in the incidence of cancer and the clinical stage at diagnosis. An uneven distribution of specialists and facilities, lack of standardized treatment, and inadequate provision of clinical training might explain the difference in choice regarding gastric resection [31, 32]. We also noted the regional differences in gastroenterological specialists. For example, the number of endoscopic surgical skill qualification system–qualified gastric surgeons per million general population was 12.0 in Wakayama and 0.8 in Aomori. The proportion of open gastrectomies and laparoscopic gastrectomies was respectively 5.3% and 23.8% in Wakayama and 36.0% and 8.5% in Aomori. However, it is difficult to clarify the relationship between the regional differences in treatment and specialist. Laparoscopic gastrectomy is not exclusively performed by endoscopic surgical skill qualification system–qualified gastric surgeons. Not all board-certified fellows of the Japan Gastroenterological Endoscopy Society perform ESD. NDB Open Data does not have individual data or detailed clinical data, and our study is an ecological study. Further studies using the NCD, which contains detailed clinical information including participation of board-certified surgeon [8] or large-scale questionnaire surveys, are necessary to elucidate the relationship between regional differences in gastric resection and specialists. The establishment and dissemination of clinical guidelines is also an important factor in efforts to reduce regional disparities and standardize clinical treatment of gastric cancer [33, 34]. The Japanese guidelines for treatment of gastric cancer were first published in 2001 and the guidelines for ESD and EMR for early gastric cancer in 2014, and both have been updated several times [24, 35]. However, standardization of treatment and reduction of regional disparities by dissemination of clinical guidelines remain suboptimal.

This study has several limitations. First, patient characteristics other than age, sex, prefecture, inpatient/outpatient status, and outcome data were not available. Second, NDB Open Data contains aggregated data but not individual data. Therefore, it is not known how many patients underwent more than one endoscopic resection or how many underwent surgery after incomplete endoscopic resection. Although these numbers must have been similar to the number of gastric resections, we could not precisely calculate them. Third, as mentioned in the Methods section, some values were missing from the dataset. For example, Fig. 2 shows that all of the resections performed in the 25–29-year age group was ESDs. The only data available for this age group were for 10 ESDs performed in women, and it is unknown whether other types of resection were performed in fewer than 10 cases. However, values were missing for only 4.9% of prefectures and for 0.6% in terms of information on age and sex. Therefore, the influence of missing values is likely to be small. Fourth, no clinical or pathologic diagnoses were available. Malignant gastric tumors might include gastric lymphoma and GIST. We could not differentiate gastric resection for gastric cancer from that for GIST. Reimbursement for local gastrectomy is not limited to malignant tumors. Before 2020, ESD and LECS might have included malignant duodenal tumors. However, in this study, most gastric resections are likely to have been performed for gastric cancer because our data are similar to the number of surgeries for gastric cancer in the NCD. Fifth, there has been some inconsistency in the diagnosis of early gastric cancer and adenoma among pathologists in East Asia and their counterparts in North America and Europe [36]. Moreover, the reimbursement price for ESD in patients with gastric cancer is higher than that for EMR in those with gastric cancer and endoscopic resection for non-malignant gastric tumors. Gastric adenoma can be included in ESD for gastric malignancy in view of the differences in pathologic diagnosis and upcoding for reimbursement.

Despite these limitations, our study sheds light on the clinical epidemiology of patients undergoing resection of malignancy gastric tumors in terms of age and sex distribution, trends over time, and regional disparities. This is the first Japanese study to investigate both endoscopic and surgical resection using nationwide data. We believe that our findings will be useful for the improvement of clinical practice and the development of health care policy. Use of ESD and robot-assisted surgery has been increasing and will continue to do so in parallel with population aging and development and dissemination of novel techniques. Standardization of treatment by clinical guidelines and a more even distribution of specialists will be key to improvement of the present regional disparities in the treatment of gastric cancer across Japan. The JED database should be expanded to include nationwide clinical data on endoscopic surgery.

## Supplementary Information

Below is the link to the electronic supplementary material.

Supplementary file 1 (PDF 106 kb)

Supplementary file 2 (PDF 107 kb)

Supplementary file 3 (PDF 115 kb)

Supplementary file 4 (PDF 112 kb)

## Data Availability

The datasets used and/or analyzed in this study are publicly available.

## Abbreviations

NDB: National database of health insurance claims and specific health checkups
FY: Fiscal year
EMR: Endoscopic mucosal resection
ESD: Endoscopic submucosal dissection
LECS: Laparoscopic and endoscopic collaborative surgery
JED: Japan endoscopy database
GIST: Gastrointestinal stromal tumor
NCD: National clinical database
