# Supplementary figures and images for: Clinical epidemiology of the endoscopic, laparoscopic, and surgical resection of malignant gastric tumors in Japan, 2014–2021: a retrospective study using open data from a national claims database
Source: Gastric Cancer. 2024 Sep 28;28(1):1–11. doi: 10.1007/s10120-024-01553-y (PMC11706853; doi:10.1007/s10120-024-01553-y)

Number of gastroenterological specialists per million general population in 2024 by prefecture

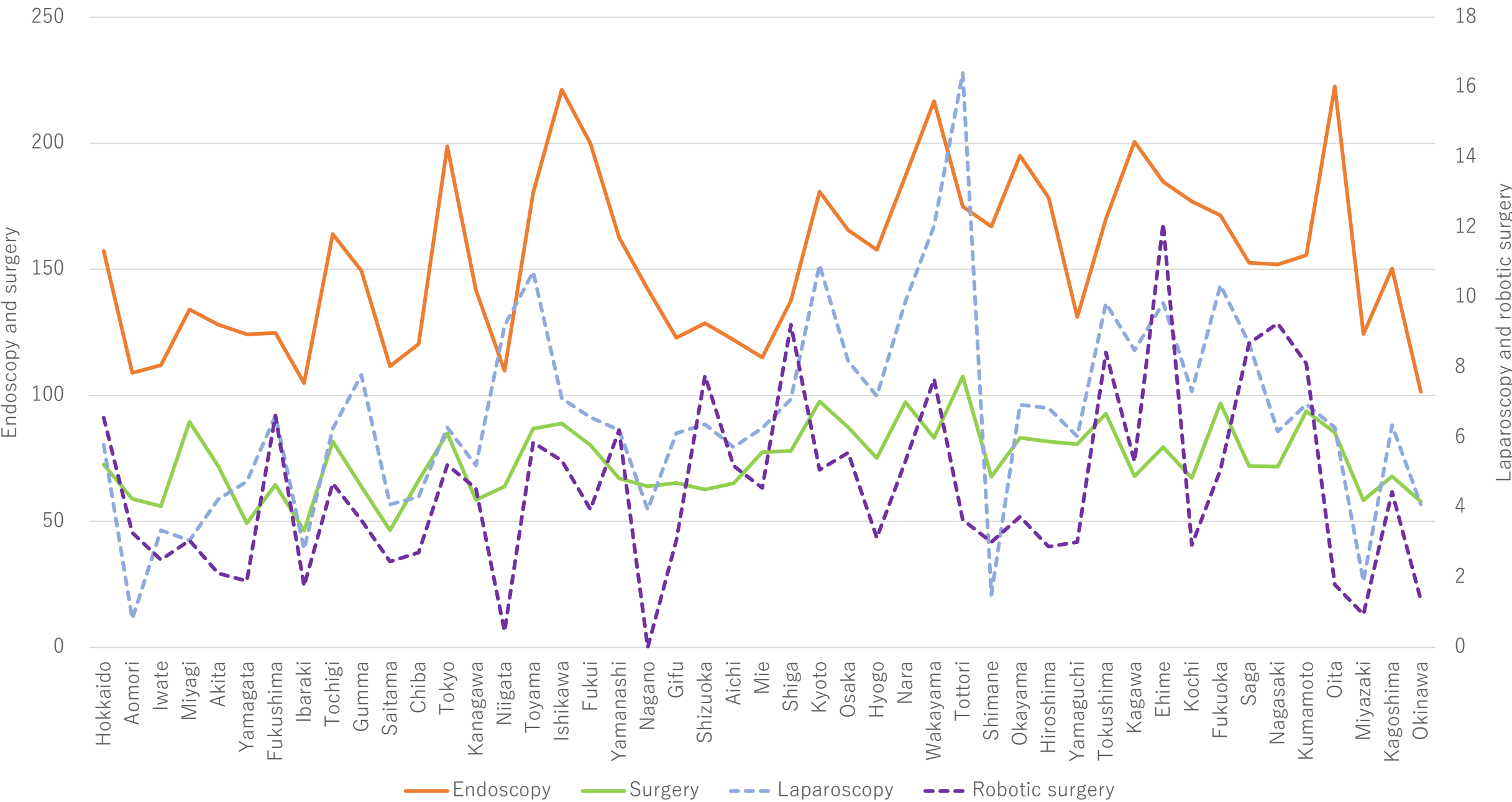

Supplement: Supplementary file 1 — (PDF 106 kb) [file 10120_2024_1553_MOESM1_ESM.pdf]

COVID-19 pandemic and monthly number of gastric resections (extent)

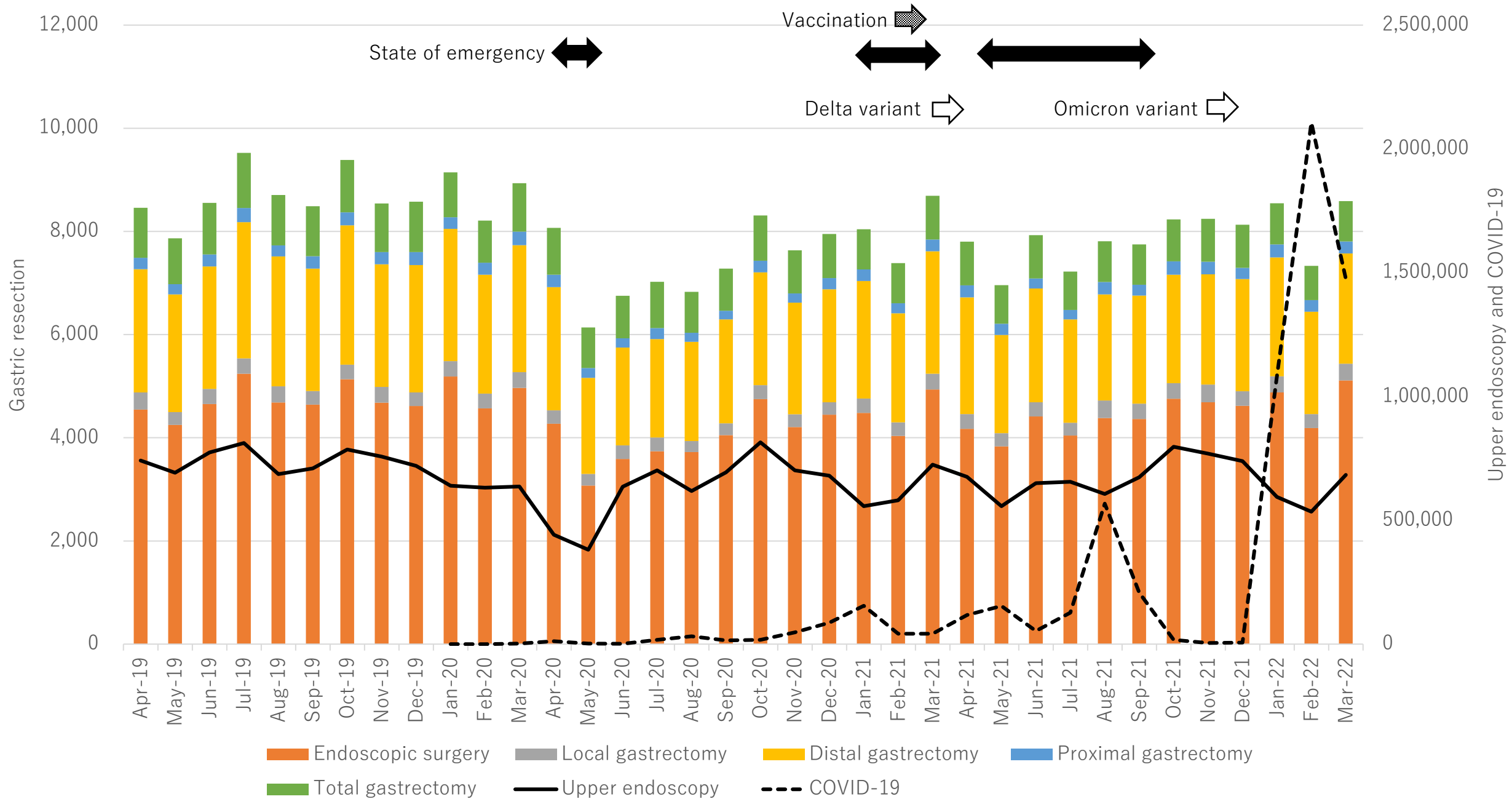

Supplement: Supplementary file 3 — (PDF 115 kb) [file 10120_2024_1553_MOESM3_ESM.pdf]

COVID-19 pandemic and monthly number of gastric resections (type)

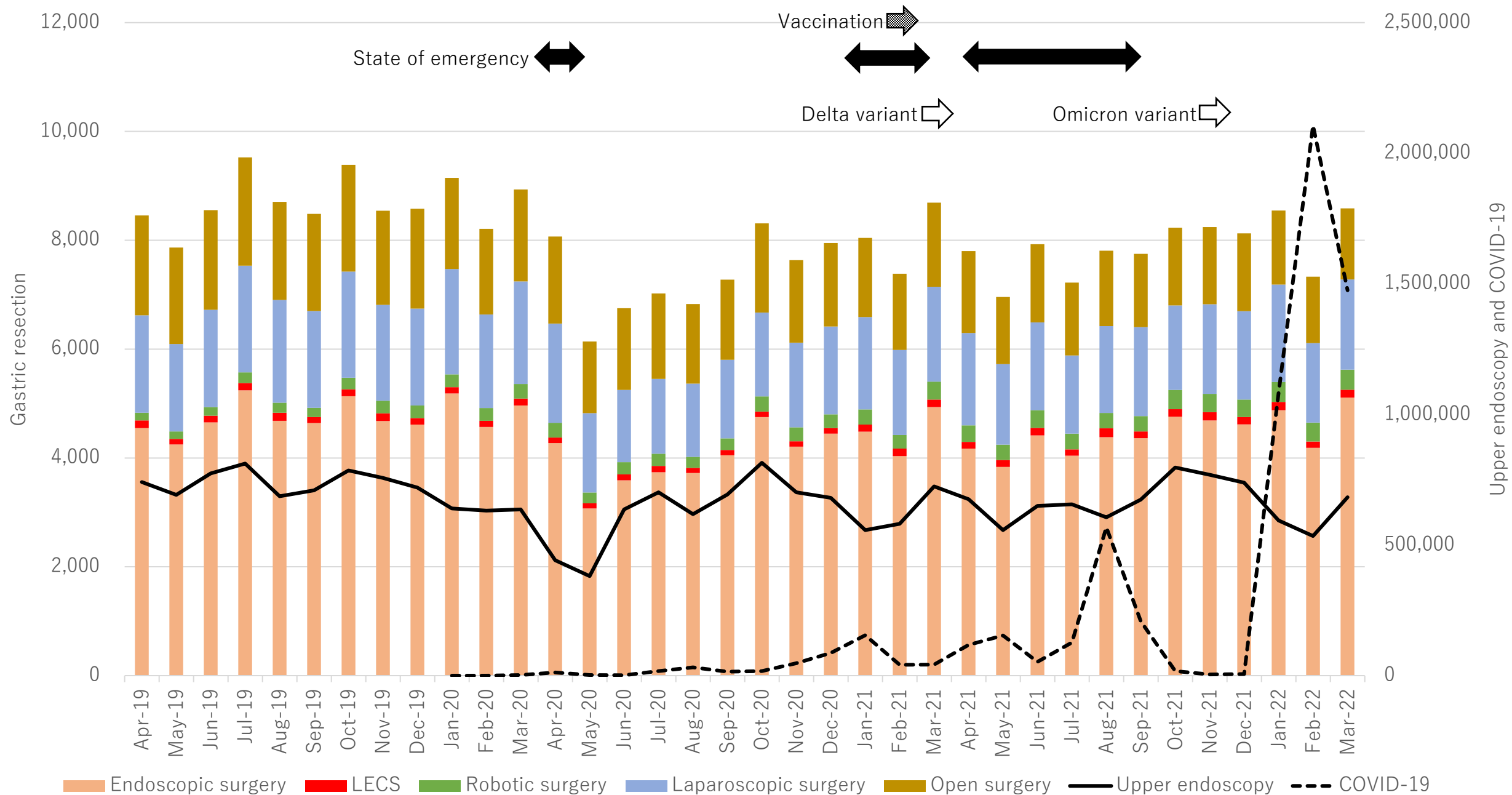

Supplement: Supplementary file 4 — (PDF 112 kb) [file 10120_2024_1553_MOESM4_ESM.pdf]
