# Supplementary material for: Clinical epidemiology of the endoscopic, laparoscopic, and surgical resection of malignant gastric tumors in Japan, 2014–2021: a retrospective study using open data from a national claims database
Source: Gastric Cancer. 2024 Sep 28;28(1):1–11. doi: 10.1007/s10120-024-01553-y (PMC11706853; doi:10.1007/s10120-024-01553-y)
Supplement: Supplementary file 2 — (PDF 107 kb) [file 10120_2024_1553_MOESM2_ESM.pdf]

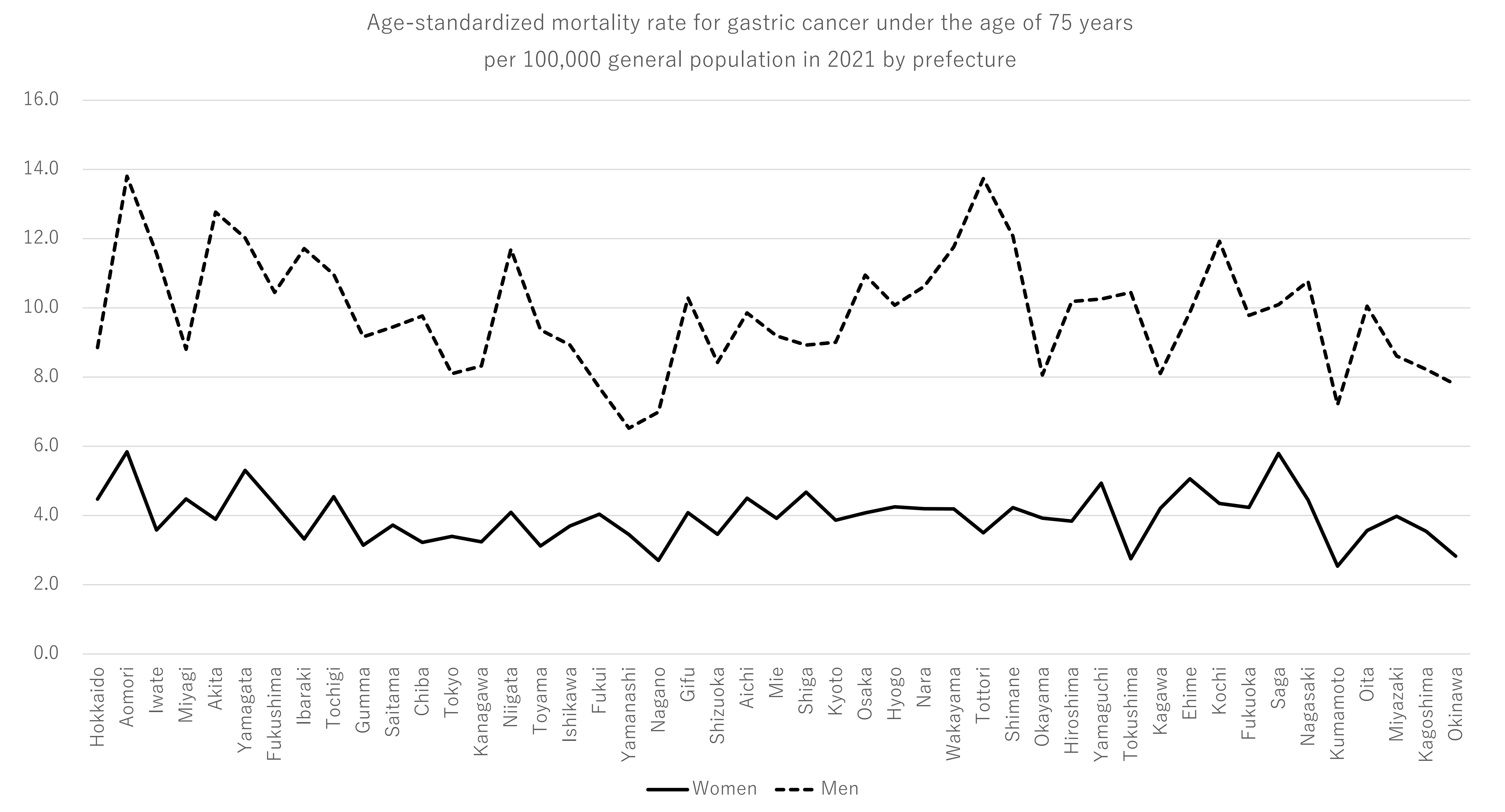
Age-standardized mortality rate for gastric cancer under the age of 75 years
per 100,000 general population in 2021 by prefecture
16.0
14.0
12.0
10.0
8.0
6.0
4.0
2.0
0.0
Hokkaido
Aomori
Iwate
Miyagi
Akita
Yamagata
Fukushima
Ibaraki
Tochigi
Gumma
Saitama
Chiba
Tokyo
Kanagawa
Niigata
Toyama
Ishikawa
Fukui
Yamanashi
Nagano
Gifu
Shizuoka
Aichi
Mie
Shiga
Kyoto
Osaka
Hyogo
Nara
Wakayama
Tottori
Shimane
Okayama
Hiroshima
Yamaguchi
Tokushima
Kagawa
Ehime
Kochi
Fukuoka
Saga
Nagasaki
Kumamoto
Oita
Miyazaki
Kagoshima
Okinawa
Women
Men
